# Simple method to detect triclofos and its metabolites in plasma of children by combined use of liquid chromatography tandem-mass spectrometry and gas chromatography-mass spectrometry

**DOI:** 10.1038/s41598-019-45790-z

**Published:** 2019-06-26

**Authors:** Hirotaka Sato, Yuki Ito, Miho Inoue, Yuki Nakahira, Satoru Hashimoto, Tamie Nakajima, Michihiro Kamijima

**Affiliations:** 1 Department of Occupational and Environmental Health, Nagoya City University Graduate School of Medical Sciences, Nagoya, 467-8601 Japan; 2 Department of Anesthesiology and Intensive Care Medicine, Kyoto Prefectural University of Medicine, Kyoto, 602-8566 Japan; 3 College of Life and Health Sciences, Chubu University, Kasugai, 487-8501 Japan

**Keywords:** Mass spectrometry, Paediatric research

## Abstract

Triclofos sodium (TCS) and chloral hydrate (CH) are widely used as sedatives for children, but no analytical method to simultaneously monitor concentrations of blood TCS, CH and their metabolites, trichloroacetic acid (TCA) and trichloroethanol (TCEOH), has been reported. The present study aimed to develop a simple analytical method for TCS and its metabolites (TCA, TCEOH and CH) in small-volume plasma from children. After acidification of specimens, TCS formic acid adduct or the metabolites derivatized using water/sulfuric acid/methanol (6:5:1, v/v) were measured by combined use of liquid chromatography tandem-mass spectrometry and gas chromatography mass-spectrometry. The limits of detection and quantification levels (µg/ml) were 0.10 and 0.29 for TCS, 0.24 and 0.72 for TCA, 0.10 and 0.31 for TCEOH, and 0.25 and 0.76 for CH, respectively. The mean recoveries were 82.8–107% for TCS, 85.4–101% for TCA, 91.6–107% for TCEOH, and 88.9–109% for CH. Within-run and between-run precision (percent of relative standard deviation, %RSD) using this method ranged from 1.1 to 15.7% and 3.6 to 13.5%, respectively, for TCS and all of its metabolites. The calibration curves were obtained with standard spiked plasma, and all of the coefficients of determination were more than 0.975. Subsequently, we applied the present method to plasma taken from five children after sedation induced by CH and TCS. In addition to TCS and CH, elevated TCA and TCEOH concentrations were detected. This new method can be applied for the pharmacokinetic analysis of TCS and its metabolites and the determination of the optimal TCS dosage in children.

## Introduction

Triclofos sodium (TCS) and chloral hydrate (CH) are widely used for procedural sedation for diagnostic imaging studies and for prolonged sedation in pediatric intensive care unit^1–3^. TCS and CH are allowed to be used as oral or intra-rectal agents to safely induce hypnosis in pediatric patients. In Japan, these sedatives are usually used in the range from 20 to 80 mg/kg for TCS and 30 to 50 mg/kg for CH. However, adverse effects have been reported, including cardiac arrhythmia^4^, respiratory insufficiency and esophagitis^5^ after overdosing (125 and 667 mg/kg of CH, respectively). Appropriate dosages of TCS and CH are needed for safer sedative protocols.

**Figure 1:**
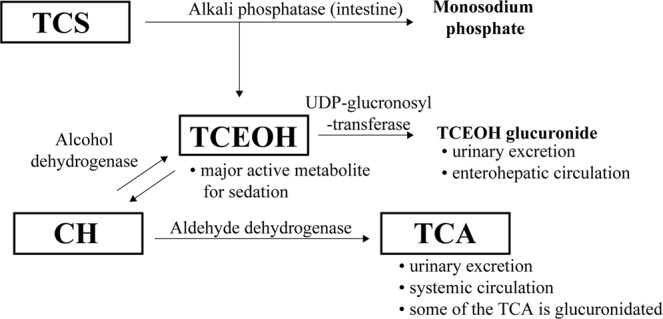
Proposed metabolic pathway of TCS and its metabolites. TCS is hydrolyzed rapidly into TCEOH and monosodium phosphate *in vivo*. The conversion of TCEOH to CH is reversible. The half-life of CH is 0.13 hours in the serum (Merdink *et al*.^6^; Bronley-DeLancey *et al*.^9^; Stenner *et al*.^7^; Henderson *et al*.^8^; Green and Prout^10^ Hembert *et al*., 1994). TCS: triclofos sodium, TCEOH: trichloroethanol, CH: chloral hydrate, TCA: trichloroacetic acid.

TCS is rapidly hydrolyzed to trichloroethanol (TCEOH), which contributes to the sedation, and monosodium phosphate (Fig. 1) after administration. Free TCEOH in the plasma is glucuronidated in the liver and excreted into the urine. Some of the TCEOH glucuronide is evacuated into bile and absorbed by the enterohepatic circulation^6,7^. CH is reversibly metabolized to TCEOH by alcohol dehydrogenase (ADH)^8^. Aldehyde dehydrogenase (ALDH) oxidizes CH to trichloroacetic acid (TCA), which is excreted into the urine^9^ and the bile^10^ as TCA glucuronide. The blood TCEOH level rapidly increases after TCS or CH administration and is eliminated from plasma with a half-life (t_1/2_) of 8.2 hours. In contrast, the plasma TCA level increases slowly and the t_1/2_ is 75.3 hours^11^. Further pharmacokinetic analyses of TCEOH and TCA are necessary in the pediatric population because children less than six months old are at higher risk of adverse events after administration of CH than older children^12^. However, there have been no reports on methods for simultaneously monitoring TCS and its metabolites in the blood.

In this study, we aimed to develop and validate a new, simple analytical method to determine TCS and its metabolites in the plasma after sedation is induced, using the combination of liquid chromatography tandem-mass spectrometry (LC-MS/MS) and gas chromatography-mass spectrometry (GC-MS).

## Materials and Methods

### Participants

Prior to sample collection from pediatric patients, written informed consent was obtained from their parents as legal guardians in the University Hospital, Kyoto Prefectural University of Medicine. The protocol of this study was reviewed and approved by the medical ethics committee of the Kyoto Prefectural University of Medicine and the ethical review committee of the Nagoya City University Graduate School of Medical Sciences (approval no. ERB-C-289 and 1112, respectively). This study was carried out in accordance with the Helsinki Declaration, and the Ethical Guidelines for Medical and Health Research Involving Human Subjects established by the Ministry of Education, Culture, Sports, Science and Technology and the Ministry of Health, Labour and Welfare in Japan. The inclusion criteria of the participants were: (i) under 12 years old with body weights >2 kg; (ii) receiving treatment in the pediatric intensive care unit for 3 days or more; and (iii) having a catheter placed in the central vein or artery for treatment purposes, which enabled non-invasive blood sampling. Patients with (i) allergy to TCS or/and CH, or (ii) cardiovascular collapse due to hemorrhage or severe cardiac dysfunction were excluded.

### Chemicals and reagents

TCA (molecular biology grade) and CH (1st grade) were purchased from Wako Pure Chemical Industries (Osaka, Japan). Dichloroacetic acid di-deuterium (DCA-d_2_), used as an internal standard (IS), and TCEOH were obtained from Sigma-Aldrich (St Louis, MO, USA) and triclofos sodium (Tricloryl^®^ syrup 10%) was from Alfresa Pharma Corporation (Osaka, Japan). Methanol (LC/MS grade), ultra-pure water (LC/MS grade), hexane (high-performance liquid chromatography (HPLC) grade), ethyl acetate (pesticide residue grade), sulfuric acid (analytical grade), formic acid (LC/MS grade), and ammonium formate solution (1 mol/l; HPLC grade) were purchased from Wako Pure Chemical Industries. Sulfatase from *Helix pomatia* (type H-2), including glucuronidase activity, was obtained from Sigma-Aldrich.

### Standard preparation and analytical procedure

Each standard was prepared in water (at a concentration of 6.0 mg/ml for TCEOH, 3.0 mg/ml for TCS and 1.5 mg/ml for TCA and CH). The pooled plasma from three healthy volunteers (written informed consent was obtained), who were neither treated with any drugs nor exposed to chemicals before the sampling, were spiked with standard solutions and used for the basic methodological examination in this study.

**Figure 2:**
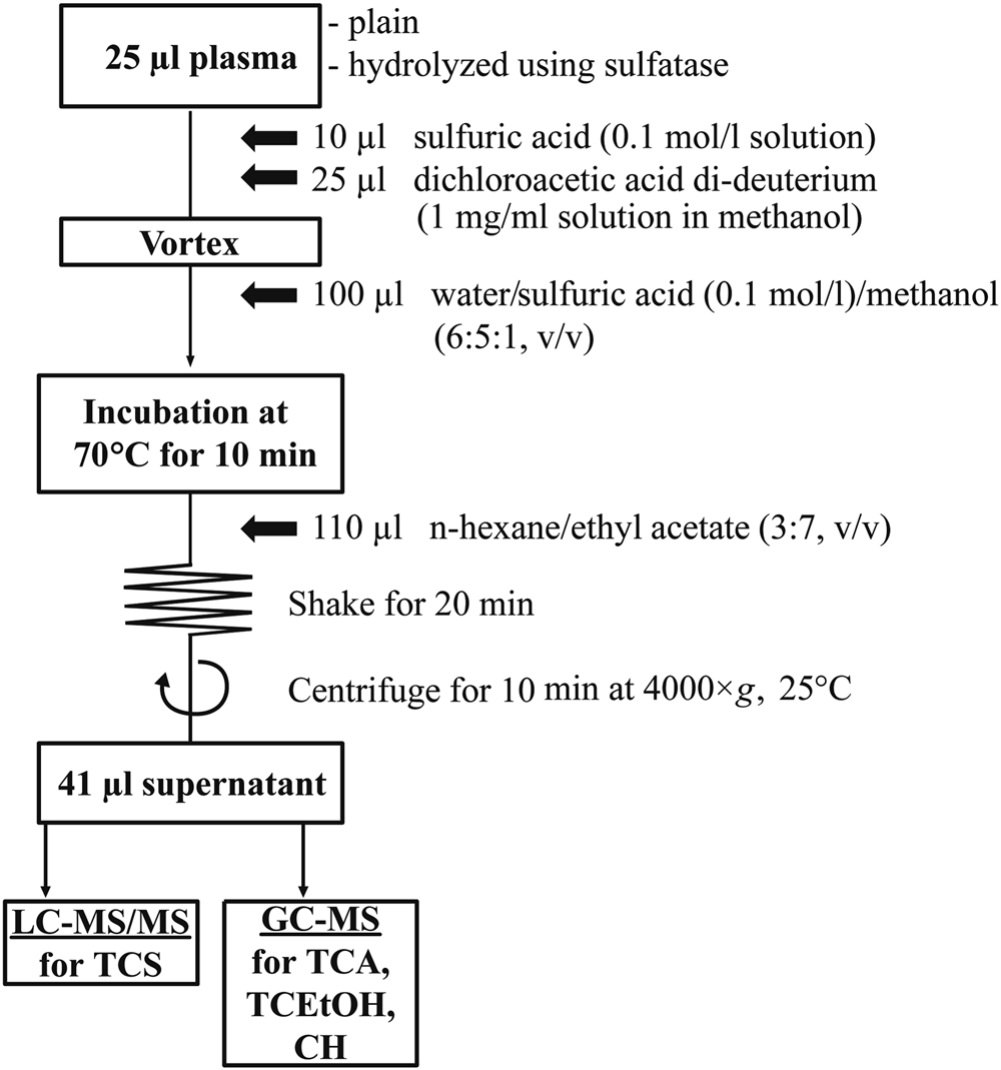
Analytical procedure for serum TCS and its metabolites. The analytes were measured by combined use of two types of mass spectrometers. TCS, triclofos sodium; TCA, trichloroacetic acid; TCEOH, trichloroethanol; CH, chloral hydrate.

The procedure for determining plasma TCS and its metabolites is shown in Fig. 2. The analytical method makes it possible for the first time to measure all the analytes with the same sample solution. The pretreatment for TCEOH, TCA and CH was based on the previously published methods^6^. Twenty-five microliters of the spiked plasma were pipetted into an Eppendorf tube, and 10 µl of sulfuric acid (0.1 mol/l) and 25 µl of DCA-d_2_ (1 mg/ml in methanol) were added. After vortex, 100 µl water/sulfuric acid (0.1 mol/l)/methanol (6:5:1, v/v) were added to the sample mixture and incubated at 70 °C for 10 min. Afterwards, 110 µl of n-hexane/ethyl acetate (3:7, v/v) were added, and the mixture was shaken vigorously for 20 min and centrifuged (4000 × g for 10 min at 25 °C). The organic solvent layer was transferred to vials and then used for the analyses of TCS by LC-MS/MS and the analyses of the metabolites (TCA, TCEOH, and CH) by GC-MS (injection volume was ten and one microliters, respectively).

### Analytical conditions of mass spectrometries

LC-MS/MS analysis of TCS was run on a LC-MS/MS-8030 system (Shimadzu, Kyoto, Japan) composed of a solvent delivery device (LC30AD), an auto sampler (SIL-30AC), a system controller (CBM-20A) and a column thermostat (CTO-20A). The LC-MS/MS conditions were as follows. The LC column was an ACQUITY UPLC^®^ HSS T3 column (Waters Corporation, Milford, MA, USA) with 2.1 × 100 mm i.d. and 1.8 µm silica. Mobile phases were 10 mmol/l ammonium formate and 0.2% formic acid (A, pH 3.1), and 10 mmol/l ammonium formate in methanol (B). Total flow rate of the mobile phase was 0.2 ml/min. Total run time including equilibration was 10 minutes. The initial mobile phase composition was 95% mobile phase A and 5% mobile phase B. The percent of mobile phase B was increased linearly over the next 5 minutes to 80%, held for 1 minute, and then increased linearly up to 95%. After that, the percentage was held for 1 minute, and the mobile phase composition returned to the initial condition where it was held for 2 minutes. The injection volume was 10 µl. The MS/MS was operated with an electrospray ionization source in the negative ion mode with multiple reaction mode. The nebulizer nitrogen gas flow was set at 1.5 l/min with the source temperature of 350 °C and drying nitrogen gas flow at 15.0 l/min. The temperatures of the desolvation line and heat block were 150 °C and 100 °C, respectively.

Analyses of plasma TCA, TCEOH and CH derivatized with methanol were performed using an Agilent 5975 inert MSD system with 6890 N GC, 7683B series injector and auto sampler (Agilent Technologies Inc., Santa Clara, CA, USA). The GC operating conditions were as follows. The GC column was Rtx-65 (Restek, PA, USA) with 30 × 0.25 mm i.d. and 0.25 µm film thickness. Initial column temperature was 50 °C, elevated to 95 °C at 5 °C/min, and then to 240 °C (held for 2 min) at 75 °C/min. The injection port temperature was 150 °C. Helium (99.999% purity) carrier gas was used with a flow rate of 1 ml/min. The injection volume was 1 µl (splitless). The temperatures of the MSD transfer line, ion source and quadrupole were 280, 230 and 150 °C, respectively. The analysis used electron ionization with 70 eV in positive ion mode. The selected ion monitoring parameters were m/z 59 for DCA-d_2_ (IS) and TCA, m/z 49 for TCEOH and m/z 61 for CH (Table 1).

**Table 1:** Compound specific mass spectrometer settings.

Compound		*m/z*		RT (min)
<LC-MS/MS>		precursor ion	product ion	
TCS		273.0	188.1	3.31
<GC-MS>		Q-ion	C-ion	
TCA		59	117	7.47
TCEOH		49	77	6.34
CH		61	83	10.11
DCA-d_2_ (IS)		59	84	5.60

Note: Chemical structure, precursor and product ions for LC-MS/MS analysis, quantification and confirmation ions for GC-MS analysis, and retention time (RT) of triclofos, its metabolites and dichloroacetic acid di-deuterium. The precursor ion for TCS was the formic acid adduct, not derivatized by methanol. C-ions and Q-ions were derived from methylated metabolites.

TCS, triclofos sodium; TCA, trichloroacetic acid; TCEOH, trichloroethanol; CH, chloral hydrate; DCA-d_2_, dichloroacetic acid di-deuterium; m/z, mass per charge ratio; Q-ion, selected ions for quantification; C-ion, selected ions for confirmation; IS, internal standard; RT, retention time.

### Assay validation

Analyses of TCS and the metabolites were carried out with an external standard method and an isotope dilution method, respectively. The linearity of the calibration curve, using pooled plasma spiked with standard solutions of TCS and its metabolites at final concentrations ranging from 0.5 to 150 or 300 (for TCEOH only) µg/ml (six or seven points), was determined by linear regression analysis. Since the concentration range of 0.5 to 75 µg/ml for TCS, TCA and CH was found to sufficiently cover the clinical concentration range and the slopes of the calibration curves were almost the same using either six or five concentration levels (data not shown), we used calibration curves derived from five levels (from 0.5 to 75) for routine analyses of these compounds. Separate calibration curves with different slopes were used to quantify high (5 µg/ml or over, three or five points) and low (5 µg/ml and below, three points) concentrations.

The limit of detection (LOD) levels were calculated from the following equations^13^: LOD = t _(0.01, n-1)_ × s; where t = Student’s *t* (one-sided), n-1 = degrees of freedom, and s = the standard deviation of seven replicates at the lowest concentration level of the calibration curve corresponding to a signal-to-noise ratio of three. The limit of quantifications (LOQ) was defined as 3 × LOD.

The within-run accuracy and precision were evaluated by spiking standards into the pooled plasma at concentrations of 0.5, 5, 25, 75, and 300 µg/ml for TCEOH and 0.5, 5, 25, and 75 µg/ml for the other analytes (n = 7). In addition, the between-run accuracy and precision were examined on seven different days with seven assay runs of the pooled plasma spiked with standards at the same concentrations adopted in the within-run assays. The percent of the nominal concentration was calculated to determine the accuracy, and the relative standard deviation (%RSD) was calculated to determine the precision.

For stability, the analyte concentration in the matrix was determined using spiked plasma samples at two concentrations (75 and 1 µg/ml for all analytes, n = 3). Plasma samples were incubated at 4 and 37 °C for 24 hours, after which TCS and its metabolites were measured. The accuracies of plasma samples incubated for 24 hours were compared with those of plasma before incubation. The stability of prepared samples injected into the GC-MS and LC-MS/MS was also tested at the same concentrations. The analytes in prepared samples were analyzed immediately and after storage for 4, 8 and 24 hours in each GC-MS (at room temperature) and LC-MS/MS (at 4 °C) autosampler. Stability was assessed by comparing the peak areas of samples at each time point within 24 hours with those of samples stored for 0 hours. Additionally, plasma samples prepared at 0 °C were compared with those prepared at room temperature.

To evaluate the matrix effect, three individual plasma samples at four concentrations (0.5, 5, 25, 75 µg/ml) were prepared and analyzed by the procedure in this study (n = 3). Carry-over in the blank sample was assessed by injecting blank samples after measuring samples with analytes at high concentrations (150 or 300 µg/ml).

### Application of the developed method to clinical samples

To examine whether the newly developed method could be used to monitor TCS and its metabolite concentrations in children’s blood, it was applied to 16 plasma samples taken from five children (one male and three females aged 3.3–4.5 months old, and another male aged 4.6 years old) who were under sedation following operations for congenital heart defects. Post-operative sedation in the pediatric intensive care unit was managed by the rectal administration of CH (31–49 mg/kg), followed by oral administration of TCS (61–79 mg/kg). Blood was collected three times (just before the TCS administration, and at about two and seven hours after that). In one participant to whom TCS was administered five times, an additional blood sample was taken before the first TCS administration. The plasma samples were obtained by centrifugation and stored at minus 80 °C until the assay.

In order to detect conjugated TCEOH in the plasma, the samples were hydrolyzed by adding sulfatase (61 units) and incubating for 40 hours, which was the optimal condition determined in this study (see Results and discussion section). Although glucuronide of TCEOH is a well-known metabolite, and a sulfate conjugate has not been documented in previous studies conducted mostly among adults^6,14^, it was reported that sulfate conjugation activities for some drugs are more active than glucuronidation in neonates and infants^15,16^. It is thus possible that conjugated-TCEOH might have included both glucuronide and sulfate in the children’s samples. Hence, we decided to use sulfatase for conjugate dissociation. The sulfatase (61 units) included 21844 units of β-glucuronidase as well because sulfatase from *Helix pomatia* has secondary activity of glucuronidase^17^.

When the concentrations were below the respective LODs, the values of LOD/square root of 2 were assigned for further calculation. Statistical analyses were conducted with EZR version 1.32 for Windows^18^.

### Ethical approval and informed consent

Written informed consent was obtained from guardians of the children participants in the University Hospital, Kyoto Prefectural University of Medicine. This study was approved by the medical ethics committee of the Kyoto Prefectural University of Medicine and the ethical review committee of the Nagoya City University Graduate School of Medical Sciences (approval no. ERB-C-289 and 1112, respectively).

## Results and Discussion

### Assay validation

The optimized parameters and retention times of analytes are shown in Table 1. The selected quantification ions for all the metabolites of TCS were the same as in previous reports^19–21^. The TCS adduct with formic acid, a precursor ion of TCS, showed the most effective transition to the product ions, whereas the other adducts such as acetic acid did not show effective transition (data not shown).

The LOD, LOQ, recovery, precision and stability are summarized in Table 2. The LOD and LOQ values were 0.10 and 0.29 µg/ml for TCS, 0.24 and 0.72 µg/ml for TCA, 0.10 and 0.31 µg/ml for TCEOH, and 0.25 and 0.76 µg/ml for CH, respectively. The within-run accuracies were 82.8–107% for TCS, 85.4–101% for TCA, 91.6–107% for TCEOH and 88.9–109% for CH in pooled plasma spiked with standard solutions of the analytes. The between-run accuracies were 95.7–103.7% for TCS, 95.1–101.8% for TCA, 89.1–102% for TCEOH and 77.9–102.8% for CH. The within-run precision ranged from 1.1 to 15.7% for TCS and all of its metabolites. The between-run precision was between 3.0 and 13.5%. The calibration curves were obtained by the standard-spiked plasma and the coefficients of determination were 0.986–0.999 for TCS, 0.975–0.992 for TCA, 0.995–0.997 for TCEOH, and 0.992–1.000 for CH (Table 2).

**Table 2:** LOD and LOQ, validation data and stability of the analytes in the matrix.

	n	Spiked concentration (µg/ml)	TCS	TCA	TCEOH	CH
LOD (µg/ml)			0.10	0.24	0.10	0.25
LOQ (µg/ml)			0.29	0.72	0.31	0.76
**R** ^**2**^ **of calibration line**						
5–75 (5–300 for TCEOH) µg/ml			0.986	0.992	0.995	1.000
LOD-5 µg/ml			0.999	0.975	0.997	0.992
**Within-run accuracy** (**% of nominal concentration**)						
	7	300			104.8	
	7	75	107.0	101.3	99.4	96.0
	7	25	95.3	96.2	104.7	88.9
	7	5	94.3	85.4	91.6	108.7
	7	0.5	82.8	97.8	106.7	104.7
**Between-run accuracy** (**% of nominal concentration**)						
	7	300			97.6	
	7	75	102.2	95.1	89.1	95.0
	7	25	103.7	98.3	92.3	77.9
	7	5	97.4	100.8	94.7	102.8
	7	0.5	95.7	101.8	102.0	99.9
**Within-run precision** (**%RSD**)						
	7	300			6.2	
	7	75	3.4	3.8	3.7	1.1
	7	25	11.8	8.7	9.1	9.0
	7	5	8.0	10.8	7.1	7.7
	7	0.5	7.4	15.7	6.1	15.4
**Between-run precision** (**%RSD**)						
	7	300			3.6	
	7	75	9.0	3.8	7.0	6.5
	7	25	11.6	3.0	13.3	12.6
	7	5	13.5	12.9	8.5	6.2
	7	0.5	12.5	3.6	5.0	9.3
**Stability of plasma sample** (**stored for 24 h**)						
4 °C	3	75	52.6	97.0	85.4	84.9
	3	1	91.6	106.5	104.5	67.4
37 °C	3	75	54.4	100.3	80.0	84.1
	3	1	93.5	110.5	96.6	72.3
**Stability of prepared sample at room temperature**						
**Hours** (**h**) **after preparation**						
0 h	3	75	87.7	92.1	109.2	91.6
	3	1	105.9	103.3	100.8	94.8
4 h	3	75	73.5	91.9	102.0	95.7
	3	1	91.6	94.5	96.9	86.8
8 h	3	75	83.7	95.8	100.6	98.5
	3	1	107.3	107.4	91.0	96.6
24 h	3	75	79.8	94.3	100.5	100.3
	3	1	99.4	106.7	87.1	87.7

Note: TCS, triclofos sodium; TCA, trichloroacetic acid; TCEOH, trichloroethanol; CH, chloral hydrate; DCA-d_2_, dichloroacetic acid di-deuterium; RSD, relative standard deviation; LOD, limit of detection; LOQ, limit of quantification; R^2^, coefficient of determination.

The stabilities of TCS and its metabolites in plasma and the prepared samples were assessed under typical storage/handling conditions. Incubation of plasma samples at 4 or 37 °C caused negligible changes in accuracy for TCA, although other analytes showed a decrement in accuracy of more than 15%. These results suggest that prompt freezing after sampling and immediate preparation after thawing are needed. TCS in plasma in particular was rapidly hydrolyzed at high concentration, although we ascertained that the preparation at 0 °C (on ice) showed approximately the same level as the nominal concentrations (data not shown). A time course analysis of the prepared samples revealed that the results measured within 24 hours were not different from those measured immediately after the preparation, except for TCS (at 75 µg/ml). However, since this analytical method was reliable for the low concentration of TCS, it was thought that TCS of the clinical samples in this study were well quantified because the maximum concentration of TCS was approximately 5 µg/ml.

The average %RSD of the matrix effect within the concentration range of 0.5–75 µg/ml was 13.9% for TCS, 5.6% for TCA, 8.9% for TCEOH and 14.9% for CH (detailed data not shown). In a carry over test, no analytes were detected in the blank sample.

The present study successfully quantified blood TCS concentrations for the first time. The LODs of the three TCS metabolites, which were measured along with TCS, were in orders similar to those reported in a previous study^22^. Reported LOD values of TCEOH and TCA in human plasma are 0.12 and 0.22 µg/ml^6^, and 0.06 and 0.04 µg/ml^19^, respectively. For CH, the LOD in rat and mouse sera has been reported to be 0.2 µg/ml^23^. TCEOH concentrations in the blood after single administration of TCS (22 mg/kg) and CH (15 mg/kg) were reportedly 8.2 and 8.5 µg/ml, respectively^11^. Since both sedatives are usually used in the range from 20 to 80 mg/kg for TCS and 30 to 50 mg/kg for CH in Japan, we consider that the present method may make it possible to analyze the plasma concentrations of TCS and its metabolites (TCA, TCEOH and CH) in the course of sedative treatment.

### Optimized hydrolyzing procedure for conjugated-TCEOH

**Figure 3:**
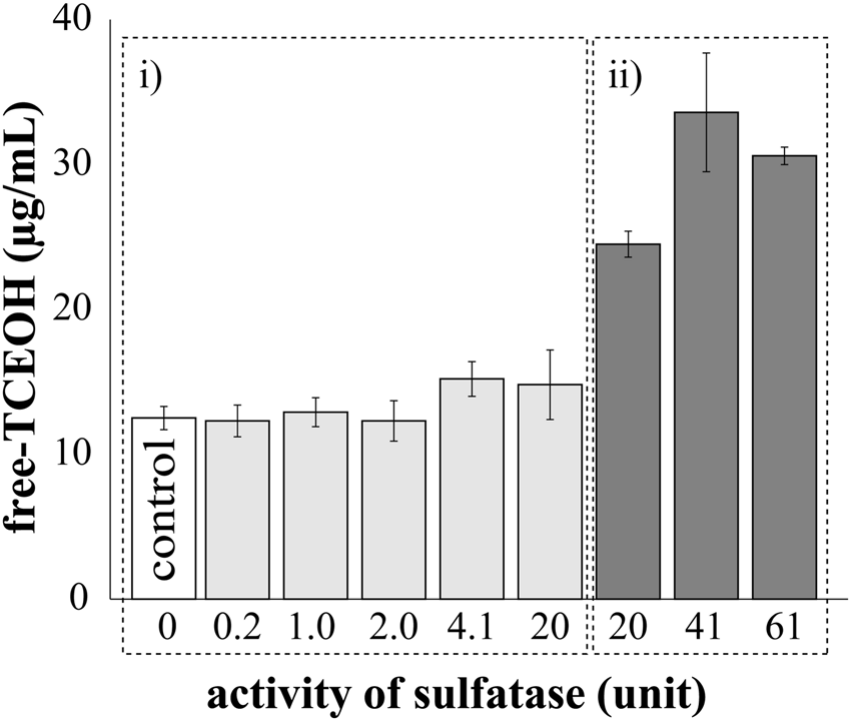
Comparison among the different hydrolysis procedures with sulfatase. The hydrolysis conditions were examined according to activity of sulfatase (0, 0.2, 1.0, 2.0, 4.1, 20, 41 or 61 units) and incubation time, (i) 16 hours or (ii) 40 hours. Each group consisted of three samples. Error bars depict standard deviations. The hydrolysis condition was assessed by free-TCEOH production (grey and dark grey bars).

Since the hydrolyzing procedure using sulfatase should be optimized, free TCEOH concentrations yielded after different procedures were investigated. Sixteen-hour incubation (Fig. 3i) was not sufficient for complete hydrolysis, judging from the results of 40-hour incubations (Fig. 3ii). Among the 40-hour incubation groups, 41 and 61 units of sulfatase yielded almost the same amount of free TCEOH in the plasma, which was apparently larger than that yielded by 20 units. The values were also larger than those of control samples measured after 16-hour incubation. To assess the hydrolysis efficiency, we set the amount of sulfatase at 61, 91.5, 122 and 183 units, followed by incubation at 40 °C. It was confirmed that with 61 units of sulfatase the conjugation was completely dissociated since the amount of free TCEOH plateaued with the addition of 61 units or more (detailed data not shown). The coefficient of variation in the hydrolyzing condition using 61 units of sulfatase was 1.9%, whereas that after incubation with 41 units was 12.2% (detailed data not shown). From this we decided to use 61 units of sulfatase in the present study.

The sulfatase included 21844 units of β-glucuronidase. Deconjugation achieved with 224 units of commercial β-glucuronidase obtained from Sigma-Aldrich (St Louis, MO, USA; including 1.6 units of sulfatase activity) was equivalent to that achieved with the above sulfatase under incubation for 40 hours (data not shown). Though it was not clear whether TCEOH sulfate existed in the blood, this optimized method using the sulfatase was considered to hydrolyze both glucuronide and sulfate conjugates (i.e. total amount of TCEOH). Based on the above results, we determined the optimum hydrolyzing condition to be a 40-hour incubation at 37 °C using 61 units of the sulfatase.

### Application of the developed method to clinical samples

**Figure 4:**
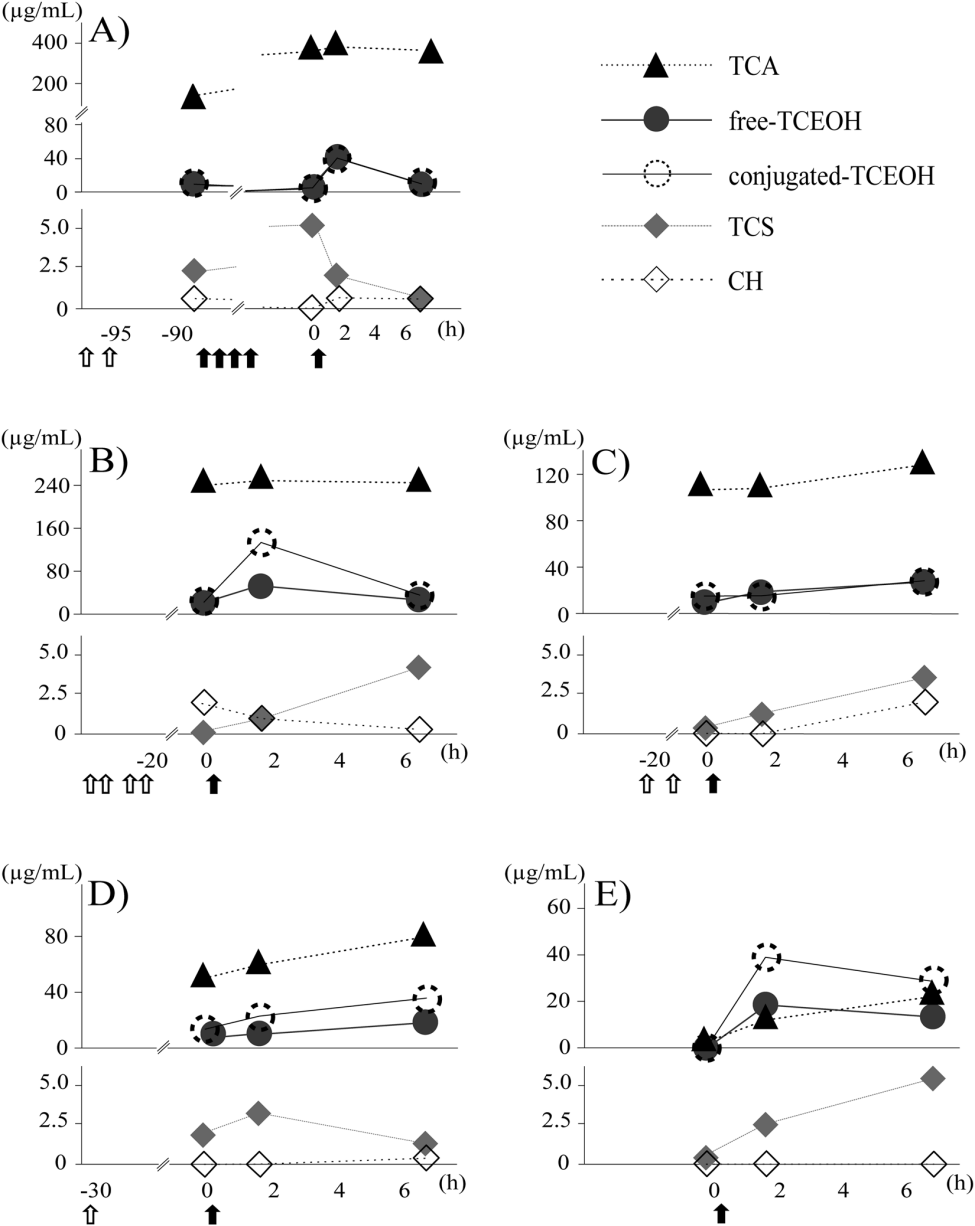
Time-plasma concentration curves of TCS and all the metabolites in the children’s samples. The clinical samples were collected from children who were in courses of sedation. The horizontal axis represents the time before/after the last TCS administration. The upper vertical axis represents the concentrations of TCA and TCEOH, and the lower one represents those of TCS and CH. Administration of CH and TCS is indicated by arrows in white and black, respectively. (**A**) 4.0-month-old female, (**B**) 3.3-month-old male, (**C**) 4.2-month-old female, (**D**) 4.5-month-old female and E) 4.6-year-old male. The administered doses (mg/kg) of CH and TCS for sedation each time were, (**A**) 31 and 61, (**B**) 43 and 68, (**C**) 49 and 79, (**D**) 39 and 77 and (**E**) none and 69 at the point of each arrow, respectively. The elapsed times (hours) from CH premedication to TCS administration were (**A**) 7.5, (**B**) 20.6, (**C**) 18.7 and (**D**) 30.7.

The measurement results of TCS and the metabolites in plasma taken over time from five children under sedation in a pediatric department are shown in Fig. 4. The first blood was collected just before the TCS administration (or before the last one when it was administered multiple times). CH had already been administered (Fig. 4A–D) or had not been administered (Fig. 4E) before the first blood sampling. Elevations of TCEOH (free and conjugated) and TCA concentrations after TCS administration were detected in all five children. The peak time of TCEOH was 1.6 to 7.1 hours after the last TCS administration, and TCA concentrations rose as time passed (Fig. 4A,D,E), although such increases are not clear in Fig. 4B,C. A possible explanation for this is that the children in Fig. 4B,C were administered CH multiple times (four and two, respectively) and almost 20 hours had elapsed before the first blood collection, at which time TCA concentrations were almost maximized due to the repeated dosing and its long biological half-life^11^. The child in Fig. 4D was administered CH only one time and 30.7 hours had elapsed by the first sampling, which might have been the reason why TCA concentration increased after TCS dosing.

CH was detected in the first blood collection in two subjects (Fig. 4A,B), whereas in the other two (Fig. 4C,D) CH was below the LOD. This could be explained by the fact that the children in Fig. 4A,B received multiple doses of CH as premedication, which could have led to the CH accumulation. The child in Fig. 4C also received CH loading, but CH was not detected, possibly because of a longer elapsed time than in the child in Fig. 4A. These results were not in agreement with a previous report in which the half-life of blood CH was 0.13 hours^24^ after 40 mg/kg dosing and CH was almost cleared from children’s plasma between 15 and 30 min after a single administration of 50 mg/kg^8^. In the present study, multiple dosing might have resulted in the extended biological half-life. CH was also not detected in one patient to whom CH was not administered (Fig. 4E). This child was older than others (4.6 years old versus 3.3–4.5 months old), which might have been the reason for the rapid metabolism of TCS.

In specimens taken about 2 to 7 hours after TCS sedation when TCEOH concentrations were the highest, CH was detected in four children (Fig. 4A–D). This was probably because trace amounts of CH remaining after premedication were detected in the first blood and/or the plasma CH derived from TCS (Fig. 1) was found in the plasma after TCS administration.

Overall, TCS, TCA and TCEOH were detected in 15–16 (93.8–100%) samples, and CH was detected in 8 out of 16 plasma (50.0%) taken from five children (Fig. 4). Accordingly, the present analytical method is considered to be applicable in the biomonitoring of TCS sedation.

### Advantages of this method

An appropriate method is required for small-volume blood samples for analyses of TCS and its metabolites in young children. The 25 µl of plasma needed for the present procedure meets this requirement. Additionally, the present method of sample preparation is simple and allows injection of the same pretreated specimens into LC-MS/MS and GC-MS. Only 4 hours or less are needed for sample preparation, and the necessary amount of organic solvent can be saved through the operation of analysis. Altogether, the advantages of this newly established method are the low sample volume needed and simple preparation for measurements of TCS and its metabolites.

## Conclusions

The present study succeeded in developing a reliable and simple method of detecting TCS and its metabolites in blood. This method can be applied to routine examinations for pharmacokinetic analyses of TCEOH and TCA after TCS treatment.

## References

[1] Finnemore A (2014). Chloral hydrate sedation for magnetic resonance imaging in newborn infants. Paediatr. Anaesth..

[2] Garcia Guerra G (2016). Survey of sedation and analgesia practice among canadian pediatric critical care physicians. Pediatr. Crit. Care Med..

[3] Joffe AR (2017). Chloral hydrate enteral infusion for sedation in ventilated children: the CHOSEN pilot study. Crit. Care.

[4] Han P, Song H, Yang P, Xie H, Kang YJ (2011). Cardiac arrhythmias induced by chloral hydrate in rhesus monkeys. Cardiovasc. Toxicol..

[5] Dogan-Duyar S, Willemse JL, Van Hee P, Duval EL, Neels H (2010). Chloral hydrate intoxication in a 3-month-old child: avoidance of hemodialysis by an immediate determination of trichloroethanol. Clin. Biochem..

[6] Merdink JL (2008). Kinetics of chloral hydrate and its metabolites in male human volunteers. Toxicology.

[7] Stenner RD, Merdink JL, Stevens DK, Springer DL, Bull RJ (1997). Enterohepatic recirculation of trichloroethanol glucuronide as a significant source of trichloroacetic acid. Metabolites of trichloroethylene. Drug Metab. Dispos..

[8] Henderson GN, Yan Z, James MO, Davydova N, Stacpoole PW (1997). Kinetics and metabolism of chloral hydrate in children: identification of dichloroacetate as a metabolite. Biochem. Biophys. Res. Commun..

[9] Bronley-DeLancey A (2006). Application of cryopreserved human hepatocytes in trichloroethylene risk assessment: relative disposition of chloral hydrate to trichloroacetate and trichloroethanol. Environ. Health Perspect..

[10] Green T, Prout MS (1985). Species differences in response to trichloroethylene. II. Biotransformation in rats and mice. Toxicol. Appl. Pharmacol..

[11] Sellers EM, Lang-Sellers M, Koch-Weser J (1978). Comparative metabolism of chloral hydrate and triclofos. J. Clin. Pharmacol..

[12] Heistein LC (2006). Chloral hydrate sedation for pediatric echocardiography: physiologic responses, adverse events, and risk factors. Pediatrics.

[13] US FDA. Studies to evaluate the metabolism and residue kinetics of veterinary drugs in food-producing animals: validation of analytical methods used in residue depletion studies. 2015, https://www.fda.gov/downloads/AnimalVeterinary/GuidanceComplianceEnforcement/GuidanceforIndustry/UCM207942.pdf. (Accessed 14 Dec 2018).

[14] Fisher JW, Mahle D, Abbas R (1998). A human physiologically based pharmacokinetic model for trichloroethylene and its metabolites, trichloroacetic acid and free trichloroethanol. Toxicol. Appl. Pharmacol..

[15] Levy G, Khanna NN, Soda DM, Tsuzuki O, Stern L (1975). Pharmacokinetics of acetaminophen in the human neonate: formation of acetaminophen glucuronide and sulfate in relation to plasma bilirubin concentration and D-glucaric acid excretion. Pediatrics.

[16] Alcorn J, McNamara PJ (2002). Ontogeny of Hepatic and Renal Systemic Clearance Pathways in Infants Part I. Clin. Pharmacokinet..

[17] Boppana VK, Lynn RK, Ziemniak JA (1989). Immobilized sulfatase:beta-glucuronidase enzymes for the qualitative and quantitative analysis of drug conjugates. J. Pharm. Sci..

[18] Kanda Y (2013). Investigation of the freely available easy-to-use software ‘EZR’ for medical statistics. Bone Marrow Transplant..

[19] Mudiam MK (2013). In matrix derivatization of trichloroethylene metabolites in human plasma with methyl chloroformate and their determination by solid-phase microextraction-gas chromatography-electron capture detector. J. Chromatogr. B Analyt. Technol. Biomed. Life Sci..

[20] Ramdhan DH (2008). Molecular mechanism of trichloroethylene-induced hepatotoxicity mediated by CYP2E1. Toxicol. Appl. Pharmacol..

[21] Hibino Y (2013). Sex differences in metabolism of trichloroethylene and trichloroethanol in guinea pigs. J. Occup. Health.

[22] Gorecki DK, Hindmarsh KW, Hall CA, Mayers DJ, Sankaran K (1990). Determination of chloral hydrate metabolism in adult and neonate biological fluids after single-dose administration. J. Chromatogr..

[23] Schmitt TC (2002). Determination of chloral hydrate and its metabolites in blood plasma by capillary gas chromatography with electron capture detection. J. Chromatogr. B Analyt. Technol. Biomed. Life Sci..

[24] Humbert L (1994). Determination of chloral hydrate and its metabolites (trichloroethanol and trichloracetic acid) in human plasma and urine using electron capture gas chromatography. Biomed. Chromatogr..

